# The state of leprosy: increasing trends of Hansen’s disease in Florida, USA, 1996–2025

**DOI:** 10.1371/journal.pntd.0014820

**Published:** 2026-11-06

**Authors:** Max Kevane-Campbell, Siti Setyawati Mulyono Putri, Dylan Buck, Raja Idris, Thomas A. Wichelhaus, Jorge Cervantes, Nils Wetzstein

**Affiliations:** 1 Department of Internal Medicine II, Infectious Diseases, Goethe University Frankfurt, University Hospital, Frankfurt am Main, Germany; 2 Mycobacterial Infection Research Unit (MIRU), Goethe University Frankfurt, Frankfurt am Main, Germany; 3 Institute of Medical Microbiology and Infection Control, Goethe University Frankfurt, University Hospital, Frankfurt am Main, Germany; 4 Department of Science, Valencia College, Orlando, Florida, United States of America; 5 Dr. Kiran C. Patel College of Allopathic Medicine, Nova Southeastern University, Fort Lauderdale, Florida, United States of America; 6 Molecular and Experimental Mycobacteriology, Research Center Borstel, Borstel, Germany; Marie Adelaide Leprosy Center, PAKISTAN

## Abstract

Leprosy, also known as Hansen´s disease (HD), is a chronic infectious disease which mainly affects the skin, the peripheral nerves, mucosa of the upper respiratory tract, and the eyes. According to the National Hansen’s Disease Program (NHDP), there were 225 new cases of leprosy reported in the United States of America (USA) in 2023, with Florida ranking as one of the top reporting states. Using publicly available data, we analysed the overall trend of leprosy in Florida from 1996-2025. A total of 359 confirmed cases of leprosy was reported in the state during the study period. The annual incidence rate increased from 0.01 per 100,000 population to 0.15 per 100,000 population, showing a fifteen-fold increase over the 30-year study period. With a total of 113 cases reported for the study duration, Brevard County was observed to be a hotspot for confirmed leprosy cases and locally acquired infections represented the largest category of cases with known acquisition mode.

## Author summary

Leprosy is an infectious and neglected tropical disease which primarily affects the skin, the mucosa of the upper respiratory tract, and the peripheral nervous system. Often considered as a “medieval disease”, leprosy is still widely distributed globally, including the United States of America, to this day. In this study, we analysed 30-years’ worth of data regarding confirmed leprosy cases reported in the state of Florida and analysed the overall temporal, spatial, and demographic trends observed. Results obtained from the various analyses showed that there has been a gradual increase of leprosy cases occurring in the state of Florida, with case numbers increasing from 2 cases in 1996 all the way to 36 cases in 2025. Additionally, we found that Brevard County reported the highest number of cases and locally acquired infections represented the largest category of cases with a known mode of acquisition in the 30-year period. Our overall findings emphasize that local clinicians should remain vigilant for leprosy in suspected cases, especially in areas which reported a high number of confirmed cases, as well as how public awareness should be maintained for the occurrence of locally acquired cases within the state.

## Introduction

Leprosy, also known as Hansen´s disease (HD), is a chronic infectious disease which mainly affects the skin, the peripheral nerves, mucosa of the upper respiratory tract, and the eyes [1,2]. Despite being portrayed as a “medieval disease” [3,4], the disease is still widely distributed in the world today, with an emphasis on low- and middle-income countries (LMICs) such as the Philippines, Bangladesh, Brazil, India, and Indonesia reporting from either 1,000 to over 10,000 new cases every year [1]. In high income countries, the disease is often considered absent.

Leprosy has historically been rare in the United States of America (USA), with incidences peaking around 1983, followed by a significant decline in documented cases from the 1980s to 2000 [5]. Cases are more commonly reported in the USA in states with warm, humid, and tropical or subtropical climates, particularly in the Southeastern and Gulf Coast regions [6]. The causative agents of leprosy are two slow-growing mycobacteria (SGM) species; *Mycobacterium leprae* (*M. leprae*) and–in rare cases–*Mycobacterium lepromatosis* (*M*. *lepromatosis*) [1,7]. The disease may result in severe disabilities [2] and visible deformities. The latter are a major factor which contribute to stigma, impacting both healthcare-seeking behaviour and social inclusion in infected individuals [2]. Early diagnosis and timely treatment regimens using multidrug therapy (MDT) can greatly reduce the impact of leprosy [2]. Since 1995, the World Health Organization (WHO) has provided MDT free of charge to all patients worldwide, benefiting over 17 million individuals in the past four decades [2].

The transmission of the causative agents is not completely understood, given the inability to culture the bacteria *in vitro*, however respiratory spread via nasal droplets from infected individuals with leprosy is considered to be the primary route of transmission [8]. Other potential sources of transmission can also include soil, water, and free-living amoeba as *M*. *leprae* has been isolated from said sources [8]. Further, the bacteria have been isolated from armadillos, squirrels, and primates (both in captive and wild populations) [7,8].

Recent studies have confirmed that nine-banded armadillos (*Dasypus novemcinctus*) serve as a host reservoir for *M*. *leprae* and have been implicated as a possible zoonotic transmission source of leprosy in the United States (US), particularly in Texas, Louisiana, Alabama, Georgia, and Florida [7,9,10], where they are commonly found (Fig 1) [11]. Armadillos have not been observed to act as a reservoir for *M*. *lepromatosis*, especially in populations habituating the Americas as well as in any other animal, but the mycobacterial species has been isolated in red squirrel (*Sciurus vulgaris*) populations located in Europe (i.e., Republic of Ireland, Scotland, and England) [12–14]. According to the National Hansen’s Disease Program (NHDP), there were 225 new cases of leprosy reported in the US in 2023, with Florida ranking as one of the top reporting states (all caused by *M*. *leprae*) [6,15].

**Fig 1:**
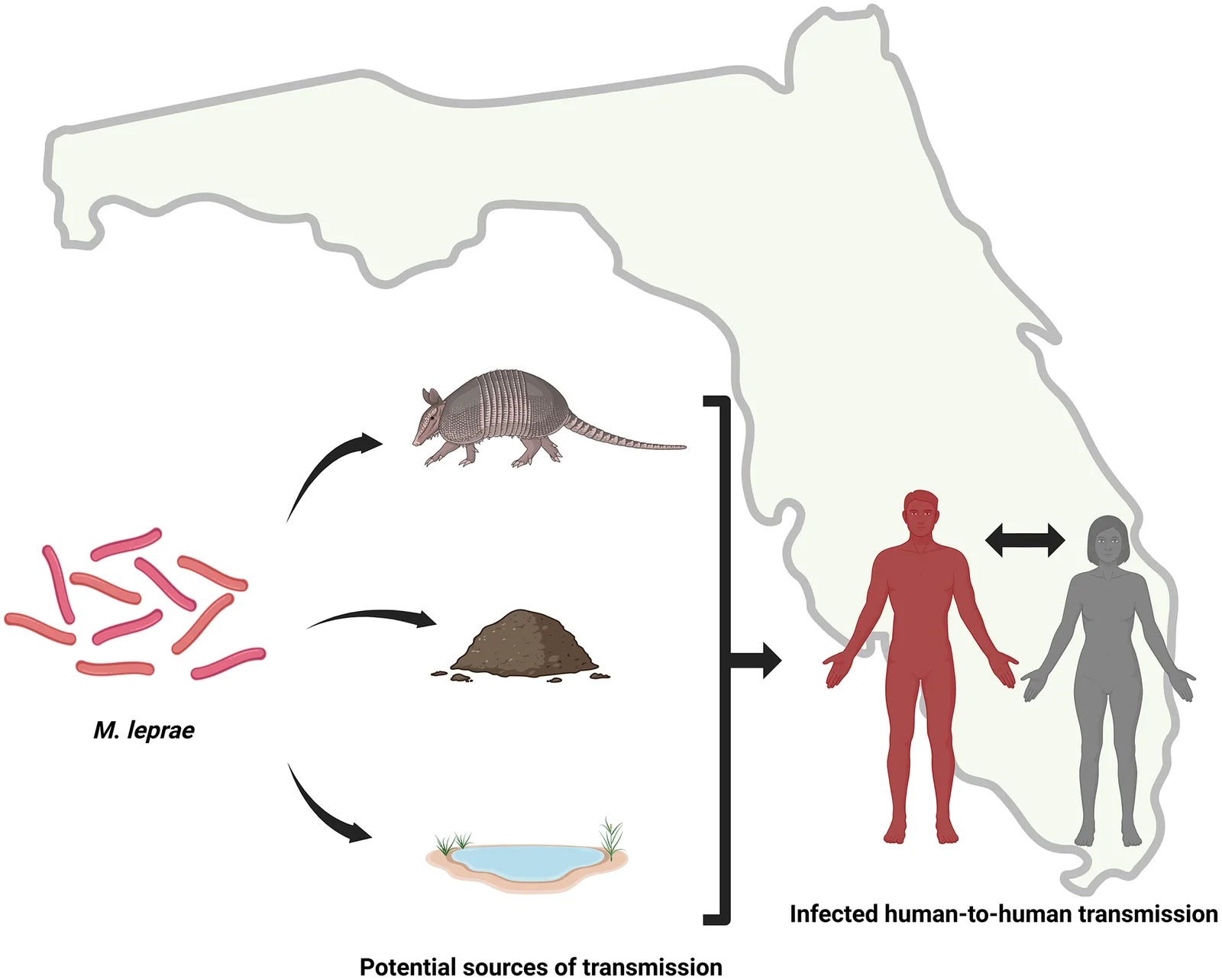
Potential sources of transmission of the causative agent of leprosy (*M*. *leprae*) in the state of Florida. Figure was created using BioRender.com.

The aim of this study was to interpret the temporal trends and identify geographical hotspots of leprosy across Florida from 1996 to 2025.

## Methods

### Analytic objectives

The analytic objectives for this study were to carry out three different analyses on the reported confirmed cases of leprosy in the state of Florida. These analyses included temporal (to observe what years reported the highest and lowest confirmed cases of leprosy in Florida and whether there is an overall increase in case numbers), spatial (to identify potential geographical hotspots), and demographic analyses (to identify age groups at risk and the most and least common form of acquisition).

### Data collection

Data on annual reported confirmed leprosy cases, affected counties, age groups (in five-year intervals from 00-04 to 85 years and above), the acquisition status of cases in Florida, as well as estimated annual total population of the state of Florida and its individual counties were obtained from the FLHealthCHARTS database from January 1^st^ 1996 to December 31^st^ 2025 (accessed on 26/08/2026) [16–18]. The database is run by the Florida Department of Health (FLDOH), Bureau of Epidemiology (BOE) [19].

Confirmed leprosy cases were identified and classified according to the 2025 Centers for Disease Control and Prevention (CDC) surveillance case definition for HD [20,21]. This characterization is based on clinical criteria, laboratory criteria, and epidemiological linkage. The CDC uses the Ridley-Jopling five-group and the WHO two-group classification systems for the identification and classification of confirmed leprosy cases [15,16].

Confirmed leprosy cases in the state of Florida were retrieved from the reportable diseases datasets of the database, being defined as cases which have occurred in the state of Florida among Florida residents. The location of confirmed cases was assigned to Florida State counties by national guidance outlines and based on the county of residence at the time of the disease identification, regardless of where they became ill or were hospitalized, diagnosed or exposed. The database assigned confirmed cases to the selected year based on the date the case was determined to have enough information to be submitted by county health department epidemiology staff to the FLDOH, BOE for state-level review. The FLHealthCHARTS database categorised the acquisition origin of confirmed leprosy cases by the following; acquired in Florida: the patient was infected within the state limits of Florida.; acquired in USA (not in Florida).: the patient was infected in another USA state and not within Florida.; acquired outside USA.: the patient was infected in a foreign country and not within the USA.; unknown.: origin of disease acquisition could not be determined. Of note, the FLHealthCHARTS database recognises Puerto Rico and the US Virgin Islands as USA territories, and these were included into the category of “acquired in the USA (not in Florida).”. Data recorded in this database was based on reportable disease information received by county and state health department staff from physicians, hospitals, and laboratories throughout the state obtained through passive and active surveillance.

Obtained data relating to statewide confirmed leprosy cases within the state of Florida were then compared to publicly available datasets of national confirmed leprosy cases within the USA and its territories per year from 1996-2025. This data was obtained from the Health Resources and Services Administration (HRSA) under the NHDP [15,22]. The NHDP included USA residents and visitors to the USA who were diagnosed with confirmed cases of leprosy while in the USA in their published datasets. Data was accessed on 19/05/2026.

As only publicly available, anonymized data was used from these databases, no ethical consent was needed.

### Data and statistical analysis

All data analyses were conducted using R statistical software (version 4.6.0) [23] within the RStudio integrated environment (version 2026.08.1 + 195 “Golden Wattle”, Posit PBC) [24]. The following R packages were used: *tidyverse* for data processing and data visualization [25]; *readxl* for importing Microsoft Excel data files [26]; *MASS* for statistical modelling [27]; *lmtest* for comparing statistical models [28]*; tigris* for obtaining US Census Bureau TIGER/Line shapefile for Florida county boundaries [29–31]; *sf* for spatial vector data handling [32]; *spdep* for spatial Empirical Bayes (EB) rate smoothing, spatial weights construction and spatial autocorrelation analysis [32]; *ggplot2* [33] and *cowplot* [34] for building figures; *patchwork* for combining multiple plots into composite figures [35]; *scales* for axes and legends formatting and labelling [36]; and *broom* for tidying statistical model output [37]. We conducted three main analytical frameworks, temporal analysis, spatial analysis, and demography epidemiological analysis.

### Temporal analysis

County-level annual leprosy incidence rates were calculated for each year from 1996 to 2025. County-level leprosy cases and population size were obtained for each year from 1996 to 2025. To assess temporal trends in leprosy incidence over the 30-year study period, negative binomial regression was applied using the generalized linear model (GLM) framework with log link function, fitted using *glm.nb* function from the *MASS* package. Negative binomial regression was selected as the primary model following formal assessment of overdispersion in the initial Poisson model. A likelihood ratio test also confirmed that the negative binomial model provides a better fit than the Poisson model (p value = 0.0016).

In the negative binomial model, population size was incorporated as a log-linked offset term to model the annual incidence rate per 100,000 population, rather than raw case count. Calendar year was included as a continuous predictor variable. Incidence Rate Ratio (IRR) per year with corresponding 95% confidence interval (CI) and p-values were produced. Statistical significance was defined as p < 0.05.

To assess potential temporal autocorrelation in model residuals, autocorrelation function (ACF), and partial autocorrelation function (PACF) correlograms were examined.

A secondary negative binomial regression analysis was conducted for cases classified as acquired within Florida, to assess the temporal trend in locally acquired leprosy incidence independently of imported cases. The same model structure was applied.

Additionally, a descriptive epidemiological analysis was conducted to compare annual leprosy cases in the state of Florida and at the national level.

### Spatial analysis

Spatial autocorrelation of leprosy cases across Florida counties was assessed using the Global Moran’s I statistic. This evaluates whether the observed spatial distribution of cases differs significantly from random expectation.

To address population heterogeneity across Florida, both Global Moran’s I and Local Indicators of Spatial Association (LISA) analyses were conducted on Spatial EB smoothed rates. EB smoothing was implemented using the EBlocal() function from the *spdep* package, with population exposure quantified as person-years, the sum of annual county-level population across the study period.

County spatial relationships were defined using queen contiguity, where counties sharing either a border or a corner were classified as neighbours, and a row-standardized spatial weights matrix was constructed accordingly.

To identify local spatial clusters and outliers, LISA was computed using Local Moran’s I, using the localmoran_perm() function with 9,999 conditional permutations and a two-sided alternative hypothesis (set.seed(123) for reproducibility). Statistical significance was assessed using permutation-based p-values at a threshold of p < 0.05.

To account for the multiple simultaneous tests conducted across all 67 counties, permutation-based p-values were adjusted using the Benjamini-Hochberg (BH) False Discovery Rate (FDR) correction.

County classification into LISA quadrant types required two criteria applied jointly. First, the quadrant of the Moran scatterplot, and secondly FDR-corrected statistical significance. The Moran scatterplot’s quadrant defined by x-axis of each county’s standardized EB smoothed rate relative to the Florida’s rate, and by y-axis of the row-standardized weighted average of neighbouring counties’ standardized EB rates. Counties with both positive on the axes and with FDR corrected p-value below 0.05, were defined as High-High hotspots, or hotspots for short; counties with both negative values as Low-Low cold spots, or cold spots for short; counties with negative x-axis but positive y-axis as Low-High spatial outliers; and counties with positive x-axis but negative y-axis as High-Low spatial outliers. All remaining counties were classified as not statistically significant. Spatial distribution maps and LISA hotspot classification maps were produced using the *ggplot2* and *cowplot* package.

### Descriptive demographics analysis

Descriptive epidemiological analysis was conducted to characterize the distribution of leprosy cases by age group and acquisition status. Age-disaggregated data were analysed within each 5-year age band. Leprosy acquisition status was classified into four categories: acquired in Florida, acquired in USA (outside Florida), acquired outside USA, and unknown. The joint distribution of age group and acquisition status was visualized as a stacked bar chart, and this analysis was performed using *ggplot2*. Additionally, comparison of cases with known acquisition source, testing the proportion acquired in Florida between hotspot counties, and the rest of the state, was conducted using Fischer’s exact test.

## Results

### Temporal analysis of leprosy in Florida from 1996-2025

A total of 359 confirmed cases of leprosy were reported in the state of Florida from 1996-2025, with the highest case numbers reported in the years 2025 (n = 36), 2015 (n = 29), and 2020 (n = 27) (Fig 2A). When compared to the HRSA-NHDP national dataset, leprosy cases from Florida accounted for 7.4% of nation-wide cases within the study period (S1 Fig), increasing from 1.3% in 1996 to 20.7% in 2025.

**Fig 2:**
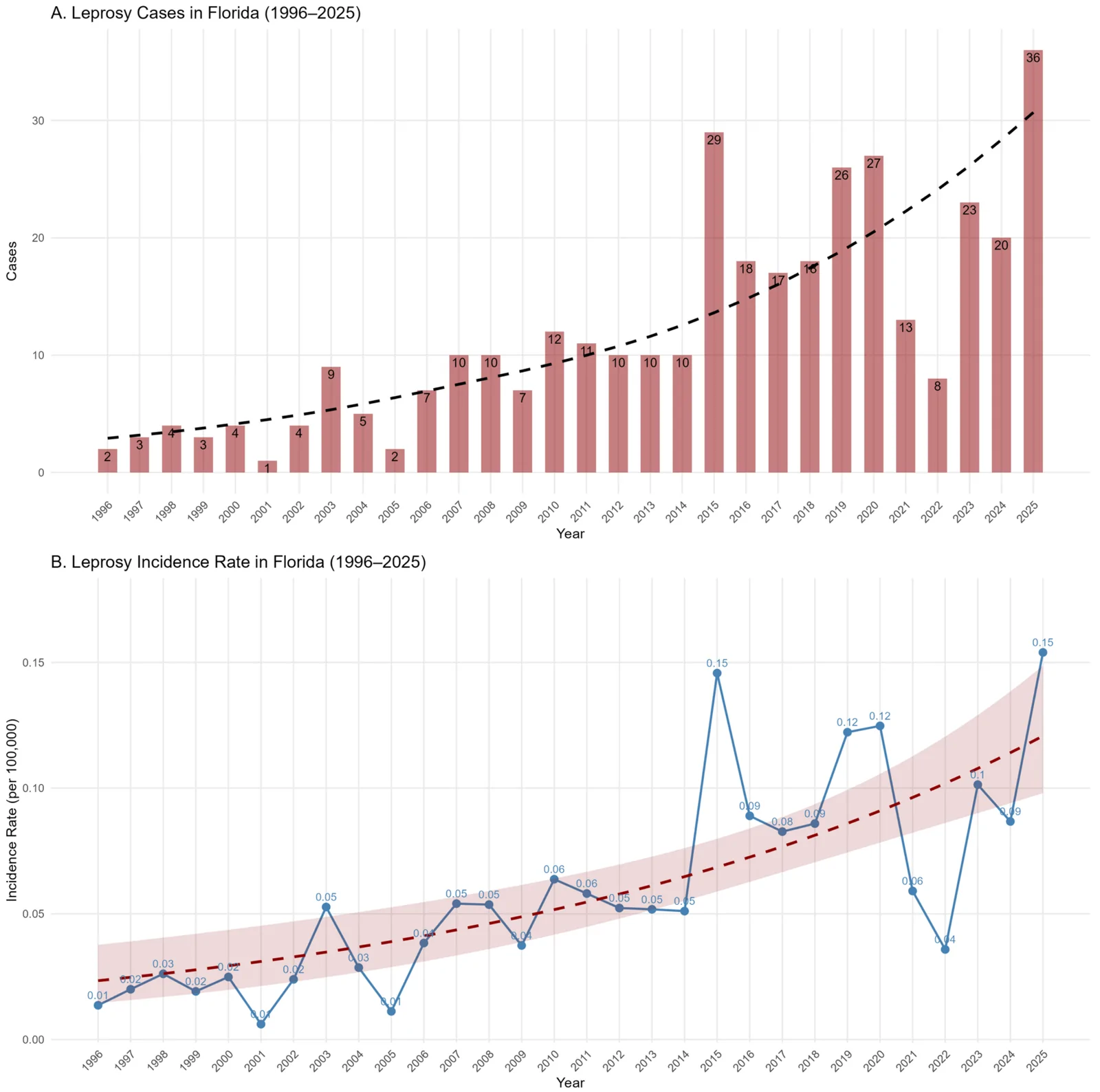
A. Confirmed leprosy cases in Florida from 1996-2025. **B.** Leprosy incidence rate in Florida from 1996-2025.

The annual incidence rate increased from 0.01 per 100,000 population in 1996 to 0.15 per 100,000 population, showing a fifteen-fold increase over the 30-year study period (Fig 2A). The highest incidence rates were recorded in 2015 and 2025, both reaching 0.15 per 100,000 population. A sudden decline was observed during 2021 and 2022, with rates falling to 0.06 and 0.04 per 100,000 population, respectively. Following this dip, incidence rebounded sharply, reaching the highest recorded rate in the final year of the study period.

Negative binomial regression analysis showed a statistically significant increasing trend in leprosy incidence over the study period. The incidence rate increased by an average of 7% per year (IRR = 1.07, 95% CI: 1.05-1.09, p < 0.0001).

Assessment of temporal autocorrelation in the negative binomial model residuals revealed no statistically significant serial correlation with no lag exceeding the 95% boundaries in either the ACF or PACF correlograms (S2 and S3 Fig).

A secondary analysis for cases acquired within Florida confirmed a statistically significant increasing trend in locally acquired leprosy incidence over the study period. The negative binomial model estimated an average annual increase of 9% in locally acquired incidence (IRR = 1.09, 95% CI: 1.06–1.13, p < 0.000001) (S4 Fig).

### Spatial analysis of leprosy in Florida from 1996-2025

Of all the counties in Florida State, Brevard County was a concentrated hotspot for confirmed leprosy cases, with a total of 113 cases reported from 1996-2025 (Fig 3A and Table 1). Brevard reported at least one confirmed case of leprosy every year since 2009. Following Brevard County were Miami-Dade (n = 32), Volusia (n = 27), Polk (n = 21), Broward (n = 17), Orange (n = 16), Hillsborough (n = 14), and Palm Beach (n = 11). All other affected counties reported 8 or less total confirmed cases from 1996-2025. Further, the average annual incidence was highest in Brevard County (Fig 3B).

**Table 1:** Overall frequency of confirmed leprosy cases and acquisition status in Florida from 1996-2025.

Frequency of Confirmed Leprosy Cases and Reported Counties in Florida from 1996-2025 [N = 359]					
Affected county/counties	Total confirmed cases n/N (%)	Acquired in Florida n/N (%)	Acquired in the USA (outside of Florida) n/N (%)	Acquired outside of the USA n/N (%)	Acquired with unknown origins n/N (%)
Brevard	113/359 (31.5%)	39/113 (34.5%)	0/113 (0%)	9/113 (8.0%)	65/113 (57.5%)
Miami-Dade	32/359 (8.9%)	5/32 (15.6%)	1/32 (3.1%)	22/32 (68.8%)	4/32 (12.5%)
Volusia	27/359 (7.5%)	13/27 (48.1%)	1/27 (3.7%)	1/27 (3.7%)	12/27 (44.4%)
Polk	21/359 (5.8%)	10/21 (47.6%)	0/21 (0%)	3/21 (14.3%)	8/21 (38.1%)
Broward	17/359 (4.7%)	1/17 (5.9%)	1/17 (5.9%)	11/17 (64.7%)	4/17 (23.5%)
Orange	16/359 (4.5%)	5/16 (31.3%)	0/16 (0%)	4/16 (25.0%)	7/16 (43.8%)
Hillsborough	14/359 (3.9%)	5/14 (35.7%)	0/14 (0%)	6/14 (42.9%)	3/14 (21.4%)
Palm Beach	11/359 (3.0%)	1/11 (9.1%)	0/11 (0%)	7/11 (63.6%)	3/11 (27.3%)
Duval	8/359 (2.2%)	2/8 (25.0%)	0/8 (0%)	1/8 (12.5%)	5/8 (62.5%)
Seminole	8/359 (2.2%)	5/8 (62.5%)	0/8 (0%)	1/8 (12.5%)	2/8 (25.0%)
Collier	7/359 (1.9%)	0/7 (0%)	0/7 (0%)	4/7 (57.1%)	3/7 (42.9%)
Osceola	7/359 (1.9%)	3/7 (42.9%)	1/7 (14.3%)	0/7 (0%)	3/7 (42.9%)
Pinellas	7/359 (1.9%)	2/7 (28.6%)	0/7 (0%)	2/7 (28.6%)	3/7 (42.9%)
St. Johns	7/359 (1.9%)	3/7 (42.9%)	0/7 (0%)	0/7 (0%)	4/7 (57.1%)
Counties with ≤ 5 cases; Alachua, Bay, Bradford, Charlotte, Citrus, Clay, Columbia, DeSoto, Flagler, Gadsden, Hernando, Highlands, Indian River, Jackson, Lake, Lee, Leon, Levy, Liberty, Manatee, Marion, Martin, Monroe, Okaloosa, Okeechobee, Pasco, Santa Rosa, Sarasota, St. Lucie, Sumter, Taylor, Union	63/359 (17.5%)	29/63 (46.0%)	1/63 (1.6%)	7/63 (11.1%)	26/63 (41.3%)
Total	359/359 (100%)	123/359 (34.3%)	5/359 (1.4%)	78/359 (21.7%)	153/359 (42.6%)

**Note:** There were no reported confirmed cases of leprosy in the counties of Baker, Calhoun, Dixie, Escambia, Franklin, Gilchrist, Glades, Gulf, Hamilton, Hardee, Hendry, Holmes, Jefferson, Lafayette, Madison, Nassau, Putnam, Suwannee, Wakulla, Walton, and Washington from 1996 to 2025.

**Fig 3:**
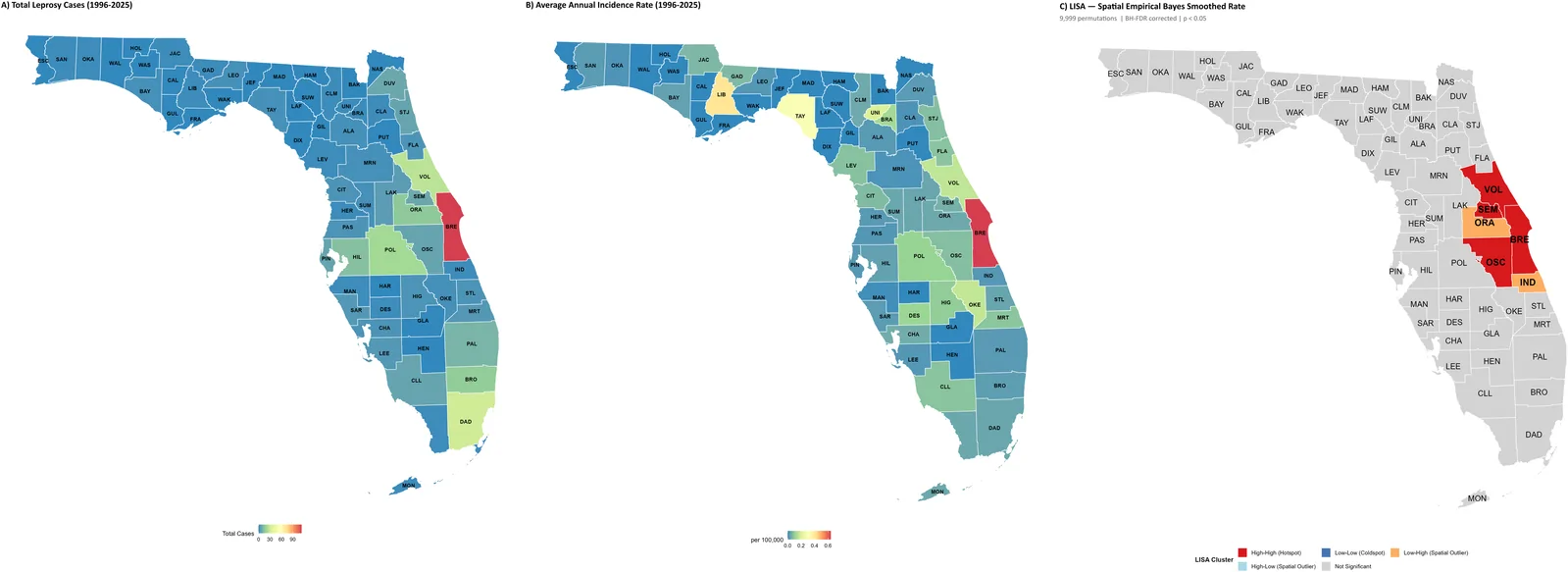
County-level leprosy burden and spatial autocorrelation in Florida (1996-2025): A. Total confirmed case counts per county. **B.** Average annual incidence rate per 100,000 population per county. **C.** Local Moran’s I Spatial Clusters. Three-letter abbreviations stand for the following Florida State counties; ALA – Alachua, BAK – Baker, BAY – Bay, BRA – Bradford, BRE – Brevard, BRO – Broward, CAL – Calhoun, CHA – Charlotte, CIT – Citrus, CLA – Clay, CLL – Collier, CLM – Columbia, DES – Desoto, DIX – Dixie, DUV – Duval, ESC – Escambia, FLA – Flagler, FRA – Franklin, GAD – Gadsden, GIL – Gilchrist, GLA – Glades, GUL – Gulf, HAM – Hamilton, HAR – Hardee, HEN – Hendry, HER – Hernando, HIG – Highlands, HIL – Hillsborough, HOL – Holmes, IND – Indian River, JAC – Jackson, JEF – Jefferson, LAF – Lafayette, LAK – Lake, LEE – Lee, LEO – Leon, LEV – Levy, LIB – Liberty, MAD – Madison, MAN – Manatee, MRN – Marion, MRT – Martin, DAD – Miami-Dade, MON – Monroe, NAS – Nassau, OKA – Okaloosa, OKE – Okeechobee, ORA – Orange, OSC – Osceola, PAL – Palm Beach, PAS – Pasco, PIN – Pinellas, POL – Polk, PUT – Putnam, STJ – St. Johns, STL – St. Lucie, SAN – Santa Rosa, SAR – Sarasota, SEM – Seminole, SUM – Sumter, SUW – Suwannee, TAY – Taylor, UNI – Union, VOL – Volusia, WAK – Wakulla, WAL – Walton, WAS – Washington. Figure was created using R statistical software (version 4.6.0) within the RStudio integrated environment 2026.08.1 + 195 “Golden Wattle”, Posit PBC packages *tigris*, for obtaining US Census Bureau TIGER/Line shapefile for Florida county boundaries, and *sf*, for spatial vector data handling. Florida county boundaries were obtained from the US Census Bureau TIGER/Line Cartographic Boundary Shapefiles (1:500,000 scale; https://www.census.gov/geographies/mapping-files/time-series/geo/cartographic-boundary.html) accessed via the *tigris* package in RStudio.

Global Moran’s I analysis indicated statistically significant positive spatial autocorrelation in the distribution of leprosy cases across Florida counties (Moran’s I = 0.1271, expected I = -0.0152, p = 0.0002), confirming that leprosy cases are geographically clustered rather than randomly distributed across the state. LISA analysis identified six statistically significant counties along Florida’s central east coast (Fig 3C). The Florida statewide mean EB smoothed rate was 0.056 per 100,000 person-years, used as the reference threshold for above- and below-average classification.

Four counties were classified as High-High hotspots, Brevard (EB rate = 0.677, FDR-adjusted p = 0.032), Volusia (EB rate = 0.179, FDR-adjusted p = 0.017), Osceola (EB rate = 0.085, FDR-adjusted p = 0.017), and Seminole (EB rate = 0.066, FDR-adjusted p < 0.001). These four counties showed EB smoothed rates above the Florida statewide mean and surrounded by neighbours whose average rates also exceeded the statewide mean. Brevard County recorded both the highest EB smoothed rate, incidence rate, and the highest number of cases of any Florida county over the study period (S1 and S2 Table).

Two counties were identified as statistically significant Low-High spatial outliers, Orange County (EB rate = 0.047, spatial lag = +1.686, FDR-adjusted p = 0.001) and Indian River County (EB rate = 0.048 per 100,000, spatial lag = +2.023, FDR-adjusted p = 0.001), both showed EB smoothed rates below the Florida statewide mean of 0.056 per 100,000, despite being geographically surrounded by counties with above-average rates. No statistically significant Low-Low cold spots or High-Low spatial outliers were detected. The remaining 61 counties showed no statistically significant local spatial clustering (FDR-adjusted p > 0.05, (S2 Table)).

### Demographic characteristics and acquisition mode of leprosy cases in Florida from 1996-2025

Within the study period, 181 out of the 359 confirmed cases (50.4%) were reported from individuals over the age of 60 (Fig 4). The number of cases peaked in the 55–59 age group followed by the 75–79 age group. Younger age groups accounted for relatively few cases. There were no confirmed cases reported below the age of 10.

**Fig 4:**
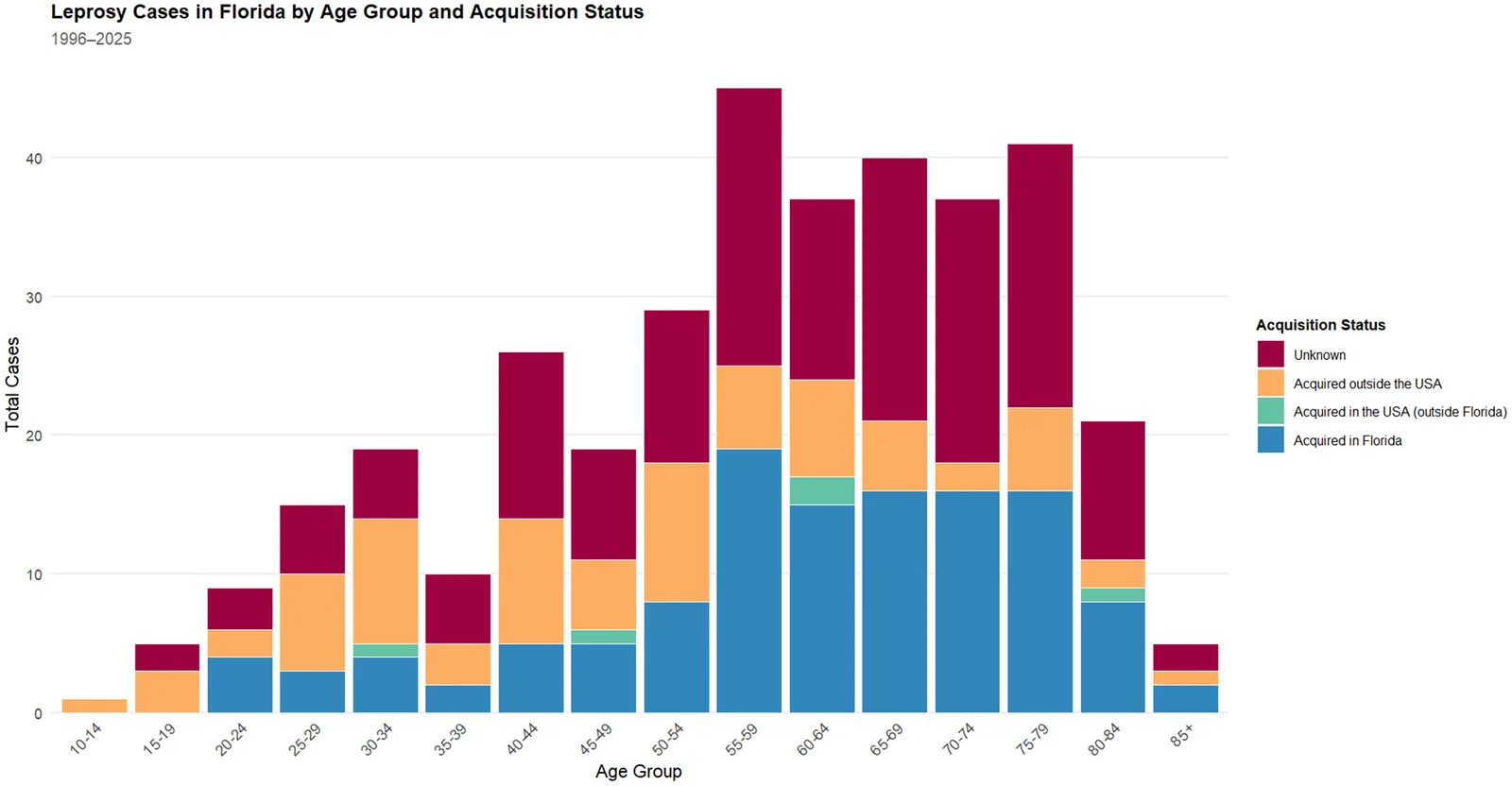
Confirmed leprosy cases in Florida by age-group and acquisition status from 1996-2025.

Out of the 359 confirmed cases, 123 cases were acquired within the state of Florida (34.3%), 5 were acquired in the USA (not in the state of Florida) (1.4%), 78 cases were acquired from outside of the USA (21.7%), and 153 cases were acquired from unknown origins (42.6%) (Fig 4 and Table 1). Among cases with known acquisition, 82.2% of hotspot-county (Brevard, Seminole, Osceola, Volusia) cases were acquired inside Florida compared to 47.4% in the rest of the state (OR = 5.09, 95% CI 2.48-11.10, p < 0.001). The main type of leprosy acquisition reported in cases with elderly individuals (individuals aged 65 years and above [38–41]) were acquisition from either an unknown source (45.3%) or in the state of Florida (40.3%).

## Discussion

This study examined the observed trends of confirmed cases as well as the incidence rate of leprosy in Florida per year from 1996 to 2025 and has shown that there is a clear annual increase of leprosy cases since 1996. Spatial analysis further identified Brevard County and its neighbouring counties as a hotspot for leprosy. Most cases with known mode of acquisition were reported to have been acquired in the state of Florida itself and not abroad. The peak incidence rate of 0.15/100,000 in 2025 is approximately ten times higher than those of other high income countries (i.e., Germany, Republic of Ireland, and Denmark with leprosy incidence rates of 0.0012/100,000, 0.019/100,000, and 0.0167/100,000 in 2024, respectively) [2,42]. The observed results provide insight to areas which report the highest number of leprosy cases—information that public health officials can use to better assess and understand the increasing incidence rate of leprosy in not just the state of Florida, but the USA in entirety and warrant future research to be conducted into these areas. It should be noted that currently there have been no leprosy cases caused by *M*. *lepromatosis* that have been reported in the state of Florida, but there have been cases reported in other parts of the US [43,44].

Central East Florida, specifically the four counties observed as hotspots, was observed to be a hotspot for confirmed leprosy cases, accounting for 43.2% of all leprosy cases in the state. This distribution aligns with this area’s humid sub-tropical climate which correlates to the persistence, transmission, and isolation of *M*. *leprae* [6,46–48]*.* In contrast to Central Florida, North Florida also possesses a sub-tropical climate but with much more frequent winter freezes and South Florida possesses a tropical climate which experiences much higher rainfall averages, primarily in the summer months [49].

As human expansion disrupts natural habitats, the likelihood of direct or indirect contact with *M*. *leprae* increases. The gradual increase of Florida’s population has led to an increase of urban infrastructure expansion, causing encroachment into rural environments where *M*. *leprae* may persist as well as regions inhabited by known zoonotic hosts, such as the nine-banded armadillo. It is hypothesized that this species of armadillos contributes to environmental contamination by burrowing and defecating—potentially introducing *M*. *leprae* into the soil, which may then serve as a transmission pathway to humans through occupational or recreational exposure [50] (Fig 1). Central East Florida has been noted to have a large nine-banded armadillo population, possibly due to the species first being introduced into Florida along the East coast in the early 1920s [51], as cumulative record sightings of the species has greatly increased in the area from 1990 to 2023 [52–54].

Out of the 14 published leprosy case reports in the state of Florida, 8 patients reported to have had previous exposure to armadillos (direct possible zoonotic transmission or exposure to soil/land that was contaminated from leprosy-infected armadillos) which helps to support this hypothesis [7]. The ongoing rise in population and urban expansion could further disturb surrounding armadillo habitats, intensifying human-wildlife interactions and possibly increasing the risk of leprosy exposure in endemic regions. Leprosy incidence rates increased 15-fold during the observation period. It could be hypothesised that this trend may be linked to increasing armadillo populations [52–54].

Climate change can extend or alter the geographic range of a pathogen host reservoir (i.e., nine-banded armadillos), either by positive or negative effects on predator populations, food supply, and other factors that can affect pathogen host reservoirs [55]. This may force the host reservoir population to extend their range to meet basic environmental needs, such as searching for water, food, or mates, or to avoid increased competition or predation from ecological changes [55]. Because of this, the number of pathogen host reservoir species, the pathological burden of individual hosts, and the chance of contact with humans (especially that of susceptible ones) could increase [55]. Climate change can also influence tropical cyclone activity, especially in Florida and the Southeast of the USA. With the rise of continued warming of both land and ocean, the increase in storm and hurricane activity is expected to occur in Florida and reach major categorical strength levels (Category 3 or higher) when they occur [56]. As the causative agents of leprosy can also be found in soil and water, it can be speculated that the disruption of the natural surrounding environment in Florida via tropical storms and hurricanes could cause large-scale mixing of water with soil and ocean water with fresh water, potentially causing a spread of water-soil *M*. *leprae* aerosolization [57,58]. Similar effects have been reported to occur with non-tuberculous mycobacteria (NTM), but not confirmed with causative agents of leprosy yet [57,58].

However, the observed surge in the number of cases might also be due to increased awareness and more accurate reporting procedures. On the contrary, a transient decline in reported cases observed in 2021 and 2022, coinciding with the COVID-19 pandemic period. This sudden decline is unlikely due to true reduction in transmission but more plausibly reflects pandemic-related disruptions to healthcare access and public health surveillance activity. This phenomenon has been observed across multiple neglected tropical diseases during this period [45]. The sharp rebound in 2023 and the attainment of highest record in 2025 further support the emerging leprosy transmission in Florida.

Cases acquired outside the US were most prominent among working-age adults, particularly those aged 25–54. This could suggest the pattern of imported leprosy among individuals with prior residence or travel history in leprosy-endemic regions. On the other hand, cases classified as acquired in Florida were distributed across nearly all age groups, supporting the evidence of autochthonous transmission. The pattern of increasing burden with age is consistent with leprosy’s characteristically long incubation period and also raises the possibility of reactivation of latent infection [59–62].

These findings collectively suggest that leprosy in Florida represents a heterogenous epidemiological picture comprising at least three speculated transmission scenarios; imported cases among working-age adults with prior travel history from endemic regions, locally acquired, potentially linked to armadillo contact across all the age groups, and possible latent reactivation of the disease among older adults due to its long incubation period.

This study has several strengths: it identifies an increasing incidence of leprosy in the state of Florida as well as geographical hotspots. However, it has also limitations: first, the true value of acquisition status is hard to obtain. It is hugely depending on the data collection methods, and the long incubation period of the disease might complicate its definition. This is mirrored by the fact that for a large number of patients this information was not available. Second, we can only provide basic demographic and no clinical data (disease severity, etc.), as this was not available from the public databases. Additionally, basic demographic data did not provide details on the sex and/or gender of infected patients. Thirdly, the data retrieved from the FLHealthCHARTS database only indicated the number of leprosy cases reported in each county but not whether infection occurred in this specific county. And finally, environmental, climatic, and reactivation hypotheses, as well as possible transmission pathways can only be hypothetically deduced from our data, as no risk exposure and behavioural data was available.

## Conclusions

The rising incidence of confirmed leprosy cases within the state of Florida in the past 30-years as well as the existence of geographical hotspots of leprosy highlights the need for more detailed monitoring of the area. Our data is consistent with possible endemic transmission. Local physicians and healthcare staff should be aware of the occurrence of leprosy in the area while the preventive measures as well as the modes of transmission require further study and research.

## Supporting information

S1 Table Overall incidence rate of confirmed leprosy cases and acquisition status in Florida from 1996-2025. (PDF)

S2 Table Florida county-level leprosy burden. Empirical Bayes (EB) smoothed incidence rates and LISA spatial cluster classification with Benjamini-Hochberg (BH) False Discovery Rate (FDR) correction (1996–2025).
(PDF)

S1 Fig A. Total number of confirmed leprosy cases in the USA from 1996-2025. B. Florida’s proportion of national confirmed leprosy cases in the USA from 1996-2025.
(PDF)

S2 Fig Autocorrelation correlogram of negative binomial model. (PDF)

S3 Fig Partial autocorrelation correlogram of negative binomial model. (PDF)

S4 Fig A. Confirmed autochthonous leprosy cases in Florida from 1996-2025. B. Autochthonous leprosy incidence rate in Florida from 1996-2025.
(PDF)

## References

[1] CDC. About Hansen’s Disease (Leprosy). Hansen’s Disease (Leprosy). 2024. https://www.cdc.gov/leprosy/about/index.html Accessed 2025 March 22.

[2] WHO. Leprosy (Hansen disease). World Health Organization. 2023. https://www.who.int/data/gho/data/themes/topics/leprosy-hansens-disease Accessed 2025 March 22.

[3] Hamilton B. The leper king and his heirs: Baldwin IV and the crusader kingdom of Jerusalem. Cambridge University Press. 2000.

[4] Wollina U, Hoenig LJ, Parish LC, Pierce SE. Leprosy in the Middle Ages: A European perspective. Clin Dermatol. 2025;43(3):431–9. doi: 10.1016/j.clindermatol.2025.01.005 39892724

[5] Bhukhan A, Dunn C, Nathoo R. Case report of leprosy in central Florida, USA, 2022. Emerging Infectious Diseases. 2023;29(8). doi: 10.3201/eid2908.220367 PMC10370849 37486691

[6] HRSA. NHDP Guide to the Management of Hansen’s Disease. 2025.

[7] Cameron S, Cervantes J. Leprosy in Florida: a rising concern. FEMS Microbiol Lett. 2025;372:fnaf122. doi: 10.1093/femsle/fnaf122 41190519

[8] Higuita NIA, Avanzi C, Henao-Martínez AF, Honap TP, Bendera N, Franco-Paredes C, et al. Leprosy. The Lancet. 2026;407:805–19. doi: 10.1016/S0140-6736(25)01963-4 41576982

[9] Truman RW, Singh P, Sharma R, Busso P, Rougemont J, Paniz-Mondolfi A, et al. Probable zoonotic leprosy in the southern United States. N Engl J Med. 2011;364(17):1626–33. doi: 10.1056/NEJMoa1010536 21524213 PMC3138484

[10] Sharma R, Singh P, Loughry WJ, Lockhart JM, Inman WB, Duthie MS, et al. Zoonotic Leprosy in the Southeastern United States. Emerg Infect Dis. 2015;21(12):2127–34. doi: 10.3201/eid2112.150501 26583204 PMC4672434

[11] NWF. Nine-Banded Armadillo. https://www.nwf.org/Home/Educational-Resources/Wildlife-Guide/Mammals/Nine-Banded-Armadillo Accessed 2025 March 24.

[12] Monsalve-Lara J, Drummond M, Romero-Alvarez D, Velho P, Jiménez-García D, Marques R, et al. Prevalence of Mycobacterium leprae and Mycobacterium lepromatosis in roadkill armadillos in Brazil. Acta Trop. 2024;258:107333. doi: 10.1016/j.actatropica.2024.107333 39067841

[13] Romero-Alvarez D, Garzon-Chavez D, Jackson M, Avanzi C, Peterson AT. Mycobacterium leprae in armadillo tissues from museum collections, United States. Emerg Infect Dis. 2023;29(3):622–6. doi: 10.3201/eid2903.221636 36823763 PMC9973711

[14] Avanzi C, Del-Pozo J, Benjak A, Stevenson K, Simpson VR, Busso P, et al. Red squirrels in the British Isles are infected with leprosy bacilli. Science. 2016;354(6313):744–7. doi: 10.1126/science.aah3783 27846605

[15] HRSA. National Hansen’s Disease (Leprosy) Program Caring and Curing Since 1894. https://www.hrsa.gov/hansens-disease Accessed 2025 March 22.

[16] FLHealthCHARTS. FLHealthCHARTS.gov: Home. 2023. https://www.flhealthcharts.gov/Charts/ Accessed 2025 March 24.

[17] Florida Department of Health. Reportable Diseases Frequency Report: Hansen’s Disease (Leprosy), 01/01/1996-12/31/2025. 2023. https://www.flhealthcharts.gov/ChartsReports/rdPage.aspx?rdReport=FrequencyMerlin.Frequency&FirstTime=True

[18] Florida Department of Health. Population Estimates Query System, 01/01/1996-12/31/2026 [Standard Report]. 1996. https://www.flhealthcharts.gov/FLQUERY_New/Population/Count

[19] Department of Health F. Hanson’s Disease. 2024. https://www.floridahealth.gov/diseases-and-conditions/disease/hansons-disease-leprosy/ Accessed 2026 June 3.

[20] Leprosy (Hansen’s Disease) 2025 Case Definition. https://ndc.services.cdc.gov/case-definitions/leprosy-hansens-disease/ Accessed 2026 June 3.

[21] CDC. Clinical Overview of Leprosy. Leprosy (Hansen’s Disease). 2025. https://www.cdc.gov/leprosy/hcp/clinical-overview/index.html Accessed 2026 June 7.

[22] New U.S. reported Hansen’s disease (leprosy) cases by year, 1985-2015. HRSA. https://www.hrsa.gov/hansens-disease/description-chart Accessed 2026 June 7.

[23] R: The R Project for Statistical Computing. https://www.r-project.org/ Accessed 2026 June 3.

[24] Posit | Data Science Platform for Enterprise Teams. https://posit.co/ Accessed 2026 June 3.

[25] Wickham H, Averick M, Bryan J, Chang W, McGowan L, François R, et al. Welcome to the Tidyverse. JOSS. 2019;4(43):1686. doi: 10.21105/joss.01686

[26] Wickham H, Bryan J, Kalicinski M, Valery K. readxl: Read Excel Files. 2026.

[27] Venables WN, Ripley BD. Modern Applied Statistics with S. New York, NY: Springer; 2002. doi: 10.1007/978-0-387-21706-2

[28] Zeileis A, Hothorn T. Diagnostic checking in regression relationships. R News. 2002;2:7–10.

[29] Walker K, Rudis B. tigris: Load Census TIGER/Line Shapefiles. 2025. doi: 10.32614/CRAN.package.tigris

[30] Bureau UC. TIGER/Line Shapefiles. Census.gov. https://www.census.gov/geographies/mapping-files/time-series/geo/tiger-line-file.html. Accessed 2026 September 11.

[31] Bureau UC. Cartographic Boundary Files - Shapefile. https://www.census.gov/geographies/mapping-files/time-series/geo/carto-boundary-file.html Accessed 2026 September 16.

[32] Pebesma E, Bivand R. Spatial Data Science. 2025. https://r-spatial.org/book/ Accessed 3 June 2026.

[33] Wickham H. ggplot2. Cham: Springer International Publishing. 2016. doi: 10.1007/978-3-319-24277-4

[34] Wilke CO. Cowplot: Streamlined plot theme and plot annotations for “ggplot2.”. 2025. doi: 10.32614/CRAN.package.cowplot

[35] Pedersen TL. Patchwork: The Composer of Plots. 2025.

[36] Wickham H, Pedersen TL, Seidel D. Scale Functions for Visualization. Posit Software, PBC. 2025. doi: 10.32614/CRAN.package.scales

[37] Robinson D, Hayes A, Couch S, Hvitfeldt E, Posit Software PBC. broom: Convert Statistical Objects into Tidy Tibbles. 2026.

[38] National Institutes of Health NIH. Age. 2025. https://www.nih.gov/nih-style-guide/age Accessed 2026 August 25.

[39] Shreya D, Fish PN, Du D. Navigating the future of elderly healthcare: a comprehensive analysis of aging populations and mortality trends using National Inpatient Sample (NIS) Data (2010-2024). Cureus. 2025;17(3):e80442. doi: 10.7759/cureus.80442 40225437 PMC11986089

[40] Singh S, Bajorek B. Defining “elderly” in clinical practice guidelines for pharmacotherapy. Pharm Pract (Granada). 2014;12(4):489. doi: 10.4321/s1886-36552014000400007 25580172 PMC4282767

[41] Himes CL. Elderly Americans. Popul Bull. 2001;56.

[42] World Health Organization O mondiale de la S. Weekly Epidemiological Record. Wkly Epidemiol Rec. 2025;100(37):365–84.

[43] Fleischmann HB, Sureshkumar N, Stratman EJ. Lepromatous leprosy infection from Mycobacterium lepromatosis without known risk factors or exposures in the Upper Midwest United States. JAAD Case Rep. 2024;45:21–3. doi: 10.1016/j.jdcr.2023.12.015 38371451 PMC10874168

[44] Olson GS, Deveaux KJ, Simmons JD, Lieberman JA. Probable Autochthonous Transmission of Mycobacterium lepromatosis in the Pacific Northwestern United States. Am J Trop Med Hyg. 2026;114(6):1078–81. doi: 10.4269/ajtmh.26-0034 42013829 PMC13235588

[45] Butala CB, Cave RNR, Fyfe J, Coleman PG, Yang G-J, Welburn SC. Impact of COVID-19 on the neglected tropical diseases: a scoping review. Infect Dis Poverty. 2024;13(1):55. doi: 10.1186/s40249-024-01223-2 39075616 PMC11285209

[46] Eichelmann K, González González SE, Salas-Alanis JC, Ocampo-Candiani J. Leprosy. An update: definition, pathogenesis, classification, diagnosis, and treatment. Actas Dermo-Sifiliográficas Engl Ed. 2013;104:554–63. doi: 10.1016/j.adengl.2012.03.028 23870850

[47] Virk A, Pritt B, Patel R, Uhl JR, Bezalel SA, Gibson LE, et al. Mycobacterium lepromatosis Lepromatous Leprosy in US citizen who traveled to disease-endemic areas. Emerg Infect Dis. 2017;23(11):1864–6. doi: 10.3201/eid2311.171104 29048278 PMC5652441

[48] Honap TP, Pfister L-A, Housman G, Mills S, Tarara RP, Suzuki K, et al. Mycobacterium leprae genomes from naturally infected nonhuman primates. PLoS Negl Trop Dis. 2018;12(1):e0006190. doi: 10.1371/journal.pntd.0006190 29381722 PMC5790234

[49] Kovacs C. Florida 101: Florida Weather. UF/IFAS Extension Flagler County. 2023. https://blogs.ifas.ufl.edu/flaglerco/2023/10/26/florida-101-florida-weather/ Accessed 2026 August 26.

[50] Liang J. Leprosy in Florida: medical experts monitoring unusual, new cases of Hansen’s disease. Emerging Pathogens Institute. 2023. https://epi.ufl.edu/2023/10/16/leprosy-in-florida-medical-experts-monitoring-unusual-new-cases-of-hansens-disease/ Accessed 2025 March 24.

[51] Florida Fish And Wildlife Conservation Commission. Land Mammals - Armadillo. https://myfwc.com/wildlifehabitats/profiles/mammals/land/armadillo/ Accessed 2026 June 9.

[52] DeGregorio BA, Deshwal A. Current Distribution of the Nine-Banded Armadillo (Dasypus novemcinctus) in the United States. Diversity. 2025;17(2):138. doi: 10.3390/d17020138

[53] Deshwal A, Degregorio BA. Current geographic distribution of nine-banded armadillos in the United States. doi: 10.5066/P1S54RPT

[54] Tarr N, Rubino M. Nine-banded armadillo (Dasypus novemcinctus) mNBARx_CONUS_2001v1 range map. 2018. doi: 10.5066/F7K073FM

[55] Liao H, Lyon CJ, Ying B, Hu T. Climate change, its impact on emerging infectious diseases and new technologies to combat the challenge. Emerg Microbes Infect. 2024;13(1):2356143. doi: 10.1080/22221751.2024.2356143 38767202 PMC11138229

[56] Florida Climate Center Office of the State Climatologist. Climate Change. https://climatecenter.fsu.edu/topics/climate-change

[57] Kambali S, Quinonez E, Sharifi A, Shahraki AH, Kumar N, Dushyantha J, et al. Pulmonary nontuberculous mycobacterial disease in Florida and association with large-scale natural disasters. BMC Public Health. 2021;21(1):2058. doi: 10.1186/s12889-021-12115-7 34758787 PMC8579656

[58] Joung YS, Ge Z, Buie CR. Bioaerosol generation by raindrops on soil. Nat Commun. 2017;8:14668. doi: 10.1038/ncomms14668 28267145 PMC5344306

[59] Carvalho AM, Almeida PS, Magalhães RG, Clara P, Silva J, Cunha J. Presumed Very late relapse of lepromatous leprosy 35 years after treatment: a diagnostic challenge in a non-endemic setting. Cureus. 2026;18(1):e101651. doi: 10.7759/cureus.101651 41705009 PMC12908493

[60] Oliveira JS de S, Reis ALMD, Margalho LP, Lopes GL, Silva AR da, Moraes NS de, et al. Leprosy in elderly people and the profile of a retrospective cohort in an endemic region of the Brazilian Amazon. PLoS Negl Trop Dis. 2019;13(9):e0007709. doi: 10.1371/journal.pntd.0007709 31479442 PMC6743788

[61] Diniz LM, Maciel LB. Leprosy: clinical and epidemiological study in patients above 60 years in Espírito Santo State - Brazil. An Bras Dermatol. 2018;93(6):824–8. doi: 10.1590/abd1806-4841.20187092 30484526 PMC6256229

[62] Sen P, Ranjan R, Middleton JR. Hansen’s disease in a native-born, United States resident, after a brief stay in an endemic area abroad. Int J Infect Dis. 2001;5(1):49–52. doi: 10.1016/s1201-9712(01)90049-9 11285160

