# Supplementary material for: The state of leprosy: increasing trends of Hansen’s disease in Florida, USA, 1996–2025
Source: PLoS Negl Trop Dis. 2026 Nov 6;20(11):e0014820. doi: 10.1371/journal.pntd.0014820 (PMC13742910; doi:10.1371/journal.pntd.0014820)
Supplement: Supplementary file 1 [file pntd.0014820.s001.pdf]

S1 Table. Overall incidence rate of confirmed leprosy cases and acquisition status in Florida from 1996-2025.

Incidence Rate and Reported Counties in Florida from 1996-2025 [N=359]					
**Affected county/counties**	**Total incidence rate**	**Acquired in Florida**	**Acquired in the USA (outside of Florida)**	**Acquired outside of the USA**	**Acquired with unknown origins**
Brevard	0.687/100,000	0.237/100,000	0/100,000	0.055/100,000	0.395/100,000
Miami-Dade	0.042/100,000	0.007/100,000	0.001/100,000	0.029/100,000	0.005/100,000
Volusia	0.179/100,000	0.086/100,000	0.007/100,000	0.007/100,000	0.080/100,000
Polk	0.115/100,000	0.055/100,000	0/100,000	0.016/100,000	0.044/100,000
Broward	0.032/100,000	0.002/100,000	0.002/100,000	0.021/100,000	0.007/100,000
Orange	0.046/100,000	0.014/100,000	0/100,000	0.011/100,000	0.020/100,000
Hillsborough	0.037/100,000	0.013/100,000	0/100,000	0.016/100,000	0.008/100,000
Palm Beach	0.028/100,000	0.003/100,000	0/100,000	0.018/100,000	0.008/100,000
Duval	0.030/100,000	0.008/100,000	0/100,000	0.004/100,000	0.019/100,000
Seminole	0.063/100,000	0.039/100,000	0/100,000	0.008/100,000	0.016/100,000
Collier	0.072/100,000	0/100,000	0/100,000	0.041/100,000	0.031/100,000
Osceola	0.082/100,000	0.035/100,000	0.012/100,000	0/100,000	0.035/100,000
Pinellas	0.025/100,000	0.007/100,000	0/100,000	0.007091/100,000	0.011/100,000
St. Johns	0.117/100,000	0.050/100,000	0/100,000	0/100,000	0.067/100,000
Counties with ≤ 5 cases; Alachua, Bay, Bradford, Charlotte, Citrus, Clay, Columbia, DeSoto, Flagler, Gadsden, Hernando, Highlands, Indian River, Jackson, Lake, Lee, Leon, Levy, Liberty, Manatee, Marion, Martin, Monroe, Okaloosa, Okeechobee, Pasco, Santa Rosa, Sarasota, St. Lucie, Sumter, Taylor, Union	0.034/100,000	0.016/100,000	0.001/100,000	0.004/100,000	0.014/100,000
Total	0.063/100,000	0.022/100,000	0.001/100,000	0.014/100,000	0.027/100,000

Note: There were no reported confirmed cases of leprosy in the counties of Baker, Calhoun, Dixie, Escambia, Franklin, Gilchrist, Glades, Gulf, Hamilton, Hardee, Hendry, Holmes, Jefferson, Lafayette, Madison, Nassau, Putnam, Suwannee, Wakulla, Walton, and Washington from 1996 to 2025.
