# Supplementary material for: The state of leprosy: increasing trends of Hansen’s disease in Florida, USA, 1996–2025
Source: PLoS Negl Trop Dis. 2026 Nov 6;20(11):e0014820. doi: 10.1371/journal.pntd.0014820 (PMC13742910; doi:10.1371/journal.pntd.0014820)
Supplement: Supplementary file 2 [file pntd.0014820.s002.pdf]

S2 Table. Florida county-level leprosy burden. Empirical Bayes (EB) smoothed incidence rates and LISA spatial cluster classification with Benjamini-Hochberg (BH) False Discovery Rate (FDR) correction (1996–2025).

County	Total Cases	Crude Rate (per 100,000)	EB Smoothed Rate (per 100,000)[1]	Standardized EB Value	Spatial Lag	Raw p-value	FDR-adjusted p-value[2]	Quadrant[3]	LISA Classification
**Brevard**	**113**	**0.6872**	**0.6772**	**7.5490**	**0.3583**	**0.0029**	**0.0324**	**HH**	**High-High (Hotspot)**
**Volusia**	**27**	**0.1792**	**0.1790**	**1.5000**	**1.1857**	**0.0012**	**0.0165**	**HH**	**High-High (Hotspot)**
Flagler	3	0.1112	0.1397	1.0226	0.5679	0.3364	0.9682	HH	Not Significant
Taylor	2	0.3119	0.1041	0.5904	-0.0379	0.9515	0.9682	HL	Not Significant
Dixie	0	0.0000	0.0991	0.5291	-0.0246	0.9751	0.9751	HL	Not Significant
Highlands	3	0.1030	0.0970	0.5045	0.0525	0.8847	0.9682	HH	Not Significant
Polk	21	0.1146	0.0953	0.4834	0.0185	0.9255	0.9682	HH	Not Significant
**Osceola**	**7**	**0.0817**	**0.0851**	**0.3598**	**1.2406**	**0.0012**	**0.0165**	**HH**	**High-High (Hotspot)**
St. Johns	7	0.1170	0.0824	0.3268	0.0755	0.8663	0.9682	HH	Not Significant
Okeechobee	2	0.1710	0.0758	0.2472	0.0364	0.9023	0.9682	HH	Not Significant
**Seminole**	**8**	**0.0627**	**0.0660**	**0.1276**	**2.2600**	**0.0000**	**0.0004**	**HH**	**High-High (Hotspot)**
Lake	5	0.0555	0.0634	0.0961	0.3107	0.4039	0.9682	HH	Not Significant
Sumter	2	0.0684	0.0618	0.0762	-0.0782	0.8588	0.9682	HL	Not Significant
Liberty	1	0.4174	0.0582	0.0330	-0.1941	0.6093	0.9682	HL	Not Significant
Calhoun	0	0.0000	0.0545	-0.0117	-0.1097	0.8070	0.9682	LL	Not Significant
Gadsden	1	0.0721	0.0525	-0.0358	-0.0922	0.8479	0.9682	LL	Not Significant
Jackson	1	0.0688	0.0522	-0.0394	-0.1684	0.6825	0.9682	LL	Not Significant
Martin	4	0.0919	0.0519	-0.0435	-0.1276	0.7778	0.9682	LL	Not Significant
St. Lucie	3	0.0369	0.0501	-0.0655	0.0376	0.9537	0.9682	LH	Not Significant
Hardee	0	0.0000	0.0492	-0.0761	0.0439	0.9184	0.9682	LL	Not Significant
**Indian River**	**1**	**0.0242**	**0.0480**	**-0.0909**	**2.0226**	**0.0001**	**0.0014**	**LH**	**Low-High (Spatial Outlier)**
**Orange**	**16**	**0.0455**	**0.0468**	**-0.1049**	**1.6860**	**0.0000**	**0.0011**	**LH**	**Low-High (Spatial Outlier)**
Union	1	0.2223	0.0455	-0.1207	-0.2659	0.5913	0.9682	LL	Not Significant
Monroe	1	0.0430	0.0455	-0.1215	-0.1988	0.7826	0.9682	LL	Not Significant
Putnam	0	0.0000	0.0453	-0.1231	0.2585	0.4797	0.9682	LH	Not Significant
Lafayette	0	0.0000	0.0448	-0.1294	0.0364	0.9320	0.9682	LH	Not Significant
Bradford	1	0.1214	0.0417	-0.1676	-0.2314	0.5987	0.9682	LL	Not Significant
Desoto	1	0.0980	0.0406	-0.1805	-0.0892	0.8364	0.9682	LL	Not Significant
Miami-Dade	32	0.0421	0.0403	-0.1840	-0.1882	0.7365	0.9682	LL	Not Significant
Citrus	3	0.0724	0.0391	-0.1990	-0.1759	0.7191	0.9682	LL	Not Significant
Lee	4	0.0216	0.0388	-0.2023	-0.2359	0.6318	0.9682	LL	Not Significant
Gulf	0	0.0000	0.0387	-0.2039	-0.1356	0.7834	0.9682	LL	Not Significant
Madison	0	0.0000	0.0381	-0.2113	-0.1375	0.7482	0.9682	LL	Not Significant
Hillsborough	14	0.0373	0.0379	-0.2140	-0.1209	0.7747	0.9682	LL	Not Significant
Collier	7	0.0722	0.0379	-0.2137	-0.2001	0.6433	0.9682	LL	Not Significant
Charlotte	3	0.0604	0.0370	-0.2239	-0.1102	0.7841	0.9682	LL	Not Significant
Broward	17	0.0318	0.0366	-0.2294	-0.2437	0.6200	0.9682	LL	Not Significant
Glades	0	0.0000	0.0355	-0.2425	-0.0422	0.9051	0.9682	LL	Not Significant
Hernando	1	0.0199	0.0346	-0.2530	-0.1509	0.7916	0.9682	LL	Not Significant
Levy	1	0.0847	0.0341	-0.2596	-0.1065	0.8009	0.9682	LL	Not Significant
Franklin	0	0.0000	0.0340	-0.2614	-0.2090	0.7149	0.9682	LL	Not Significant
Hendry	0	0.0000	0.0338	-0.2635	-0.1527	0.6637	0.9682	LL	Not Significant
Marion	2	0.0205	0.0335	-0.2670	0.1136	0.7793	0.9682	LH	Not Significant
Sarasota	4	0.0346	0.0327	-0.2767	-0.2595	0.6502	0.9682	LL	Not Significant
Duval	8	0.0301	0.0318	-0.2879	-0.1590	0.7351	0.9682	LL	Not Significant
Washington	0	0.0000	0.0316	-0.2905	-0.2882	0.5560	0.9682	LL	Not Significant
Columbia	1	0.0513	0.0314	-0.2926	-0.3055	0.4448	0.9682	LL	Not Significant
Alachua	2	0.0269	0.0311	-0.2955	-0.2186	0.5569	0.9682	LL	Not Significant
Gilchrist	0	0.0000	0.0308	-0.2999	-0.1457	0.7118	0.9682	LL	Not Significant
Bay	2	0.0399	0.0306	-0.3022	-0.1907	0.6624	0.9682	LL	Not Significant
Pinellas	7	0.0248	0.0301	-0.3076	-0.2719	0.6967	0.9682	LL	Not Significant
Baker	0	0.0000	0.0301	-0.3080	-0.2539	0.5326	0.9682	LL	Not Significant
Clay	1	0.0179	0.0300	-0.3097	-0.1119	0.7846	0.9682	LL	Not Significant

Palm Beach	11	0.0277	0.0296	-0.3138	-0.1064	0.8009	0.9682	LL	Not Significant
Pasco	3	0.0216	0.0283	-0.3299	-0.0430	0.9209	0.9682	LL	Not Significant
Jefferson	0	0.0000	0.0275	-0.3398	-0.1069	0.8221	0.9682	LL	Not Significant
Nassau	0	0.0000	0.0271	-0.3450	-0.2979	0.6671	0.9682	LL	Not Significant
Leon	2	0.0245	0.0266	-0.3507	-0.1997	0.6755	0.9682	LL	Not Significant
Manatee	1	0.0099	0.0247	-0.3741	-0.0528	0.8994	0.9682	LL	Not Significant
Hamilton	0	0.0000	0.0240	-0.3826	-0.3101	0.5889	0.9682	LL	Not Significant
Holmes	0	0.0000	0.0223	-0.4034	-0.2459	0.6643	0.9682	LL	Not Significant
Walton	0	0.0000	0.0219	-0.4078	-0.3669	0.4562	0.9682	LL	Not Significant
Suwannee	0	0.0000	0.0204	-0.4263	-0.2632	0.5461	0.9682	LL	Not Significant
Wakulla	0	0.0000	0.0179	-0.4561	-0.2297	0.6383	0.9682	LL	Not Significant
Okaloosa	1	0.0177	0.0166	-0.4717	-0.4784	0.4871	0.9682	LL	Not Significant
Santa Rosa	1	0.0216	0.0103	-0.5489	-0.5287	0.4485	0.9682	LL	Not Significant
Escambia	0	0.0000	0.0072	-0.5858	-0.5489	0.5762	0.9682	LL	Not Significant

[1] Florida statewide mean EB smoothed rate = 0.0555 per 100,000 person-years

[2] BH = Benjamini-Hochberg False Discovery Rate correction applied across all 67 counties simultaneously

[3] HH = High-High; LL = Low-Low; LH = Low-High; HL = High-Low

EB = Empirical Bayes smoothed rate per 100,000 person-years | 9,999 permutations | set.seed(123)
