# Supplementary material for: The state of leprosy: increasing trends of Hansen’s disease in Florida, USA, 1996–2025
Source: PLoS Negl Trop Dis. 2026 Nov 6;20(11):e0014820. doi: 10.1371/journal.pntd.0014820 (PMC13742910; doi:10.1371/journal.pntd.0014820)
Supplement: Supplementary file 4 [file pntd.0014820.s004.pdf]

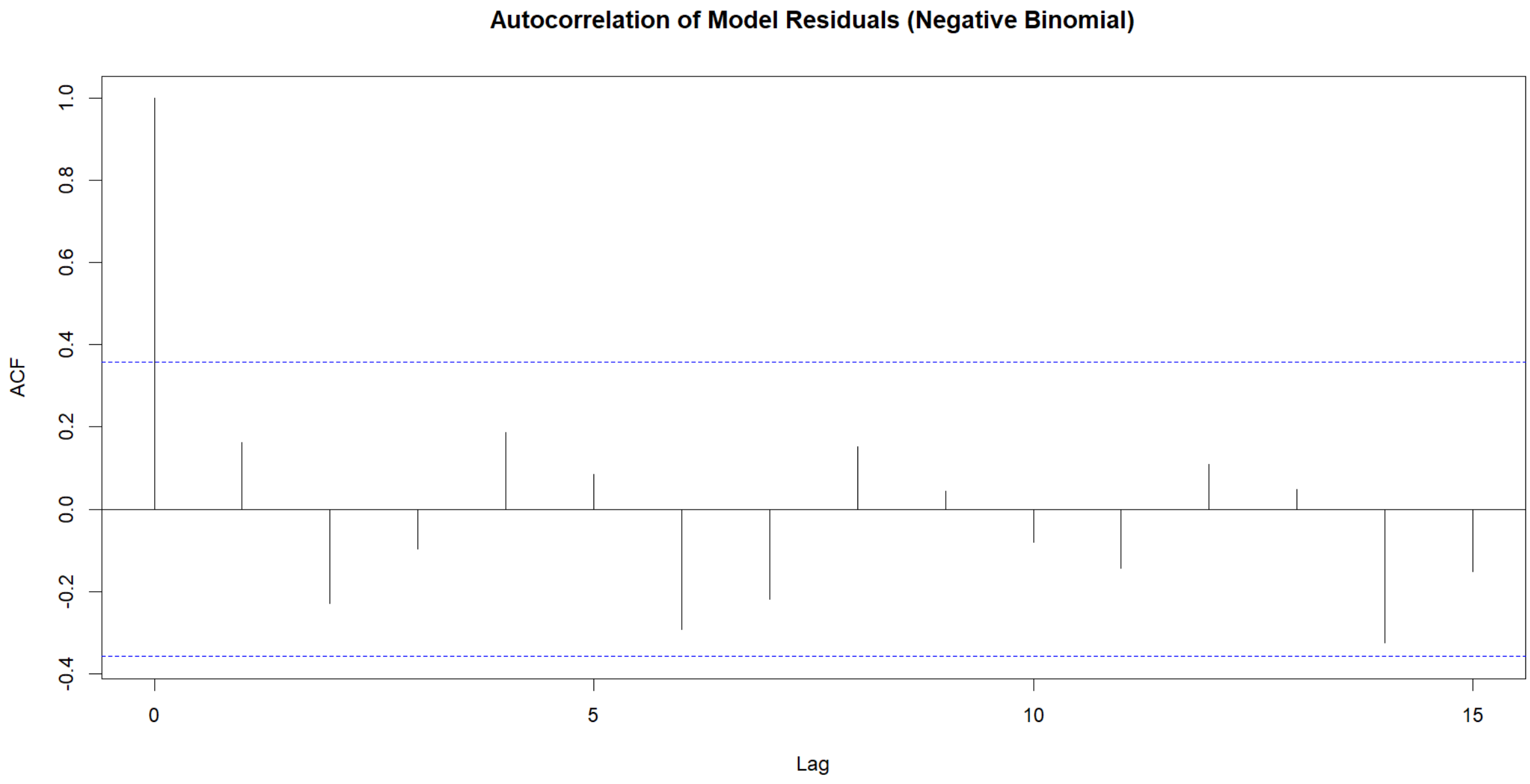

S2 Fig. Autocorrelation correlogram of negative binomial model.
